# A completely automated pipeline for 3D reconstruction of human heart from 2D cine magnetic resonance slices

**DOI:** 10.1098/rsta.2020.0257

**Published:** 2021-12-13

**Authors:** Abhirup Banerjee, Julià Camps, Ernesto Zacur, Christopher M. Andrews, Yoram Rudy, Robin P. Choudhury, Blanca Rodriguez, Vicente Grau

**Affiliations:** ^1^ Division of Cardiovascular Medicine, Radcliffe Department of Medicine, University of Oxford, Oxford, UK; ^2^ Institute of Biomedical Engineering, Department of Engineering Science, University of Oxford, Oxford, UK; ^3^ Department of Computer Science, University of Oxford, Oxford, UK; ^4^ Oxford Acute Vascular Imaging Centre, Oxford, UK; ^5^ Department of Biomedical Engineering, Washington University, St Louis, Missouri, USA; ^6^ Cardiac Bioelectricity and Arrhythmia Center, Washington University, St Louis, Missouri, USA

**Keywords:** cardiac mesh reconstruction, cine MRI, misalignment correction, electrophysiological simulation, ECGI

## Abstract

Cardiac magnetic resonance (CMR) imaging is a valuable modality in the diagnosis and characterization of cardiovascular diseases, since it can identify abnormalities in structure and function of the myocardium non-invasively and without the need for ionizing radiation. However, in clinical practice, it is commonly acquired as a collection of separated and independent 2D image planes, which limits its accuracy in 3D analysis. This paper presents a completely automated pipeline for generating patient-specific 3D biventricular heart models from cine magnetic resonance (MR) slices. Our pipeline automatically selects the relevant cine MR images, segments them using a deep learning-based method to extract the heart contours, and aligns the contours in 3D space correcting possible misalignments due to breathing or subject motion first using the intensity and contours information from the cine data and next with the help of a statistical shape model. Finally, the sparse 3D representation of the contours is used to generate a smooth 3D biventricular mesh. The computational pipeline is applied and evaluated in a CMR dataset of 20 healthy subjects. Our results show an average reduction of misalignment artefacts from 1.82 ± 1.60 mm to 0.72 ± 0.73 mm over 20 subjects, in terms of distance from the final reconstructed mesh. The high-resolution 3D biventricular meshes obtained with our computational pipeline are used for simulations of electrical activation patterns, showing agreement with non-invasive electrocardiographic imaging. The automatic methodologies presented here for patient-specific MR imaging-based 3D biventricular representations contribute to the efficient realization of precision medicine, enabling the enhanced interpretability of clinical data, the digital twin vision through patient-specific image-based modelling and simulation, and augmented reality applications.

This article is part of the theme issue ‘Advanced computation in cardiovascular physiology: new challenges and opportunities’.

## 1. Introduction

Cardiovascular diseases (CVDs) remain the principal cause of death globally according to the World Health Organization 2016 [1]. Myocardial infarction is a prominent contributor. In this condition, optimal diagnosis requires assessments of the extent, distribution and potential for recovery with cardiac interventions. However, most of the cardiac diagnostic tools, such as X-ray angiography, electrocardiography, measurement of blood biomarkers or ultrasonography, can only demonstrate the existence of myocardial ischaemia or infarction and produce limited information on its extent or stage. Cardiac magnetic resonance (CMR) imaging provides a non-invasive way to obtain images that can be combined to obtain functional and anatomical information of the heart. Because of its ability to characterize soft tissues, CMR is increasingly used to evaluate the myocardium, providing accurate assessments of left ventricular function, myocardial perfusion, oedema and scar, all of which provide important inputs in clinical decision-making [2].

Patient-specific 3D representations of the human heart are essential for the efficient realization of precision medicine. From enhancement in the interpretability of clinical data through augmented reality to comprehensive functional representations of a patient’s heart through the digital twin [3] initiative, an efficient methodology capable of producing accurate patient-specific 3D anatomical models of the human heart from CMR data is key for precision medicine applications to aid in therapeutical and diagnostic decision making. Moreover, this methodology would also contribute to the realization of *in silico* clinical trials [4] using virtual hearts accounting for the effects of anatomical variability in the human population [5].

In clinical applications, the cine magnetic resonance (MR) studies usually acquire a small number of image planes with good contrast between soft tissues at a reasonable temporal resolution, with about a typical resolution being $1\text{–}1.5\times 1\text{–}1.5\;{\text{mm}}^{2}$ in-plane and about 8–10 mm out-of-plane. CMR acquisitions are usually affected by several artefacts; in particular, the breathing misalignment from each plane being acquired at separate breath-holds. This misalignment, combined with the data sparsity caused by limited number of acquired slices, can significantly affect the accuracy of 3D reconstructions. Although significant research has been performed on 3D surface reconstruction from 2D images [6–9], a limited number has explored this problem on heart meshes, specifically from sparse 2D cine MR images.

In order to reduce slice misalignment, several methods have been introduced in the past. Some methods used slice-to-volume registration for misalignment correction [10–12], while a geometry-based approach was developed in [13] that applies iterative in-plane translations with the assumption that epicardial shapes should be smooth. One of the most common approaches for misalignment correction in heart slices is slice-to-slice registration. Using the image intensities at slice intersections, Villard *et al.* [14] optimized the similarity between intensities at the intersecting line to achieve an optimal consistency between the cross sectional intensity profiles. Some methods preferred to use a fixed slice, usually a long axis (LAX) slice, as a reference plane to align other slices [15,16].

Given a set of sparse contours or delineated curves on 2D heart slices generated by the cine protocol, methods have been developed to reconstruct geometrical heart surfaces. Zou *et al.* [17] produced a manifold interpolating surface, satisfying both geometric and topological constraints, using curves on arbitrarily oriented cross-sections. In [18,19], a cubic periodic B-spline curve was fitted to the contours, in order to uniformly sample candidate points for reconstruction of smooth 3D surface models with complex topologies. A method based on curve networks of arbitrary shape and topology on cross-section planes with arbitrary orientations was developed in [7], that maximized the data fitting while smoothing the interpolated part of the mesh. In [20,21], the authors built the 3D surface mesh from sparse, heterogeneous, non-parallel, cross-sectional, non-coincidental contours. Using a composition of smooth deformations towards the maximization of data fitting, the method provided good matching to the input data as well as visually satisfactory interpolation characteristics. A method for simultaneous misalignment and segmentation correction for cardiac images with a final 3D surface reconstruction was introduced in [22]. A deep learning-based method was proposed in [23], where the problem of mesh fitting from sparse inputs was transformed into a 3D volumetric inpainting problem followed by isosurfacing from dense volumetric data.

Although some research works that individually addressed some problems of 3D heart reconstruction from 2D cine MR images exist in the literature, a completely end-to-end automated pipeline that can generate robust and accurate 3D heart meshes, suitable for computer simulations and robust to misalignment artefacts, without any manual interaction is yet to be materialized. The objective of the present work is to develop such a pipeline and to prove that these meshes can be used in patient-specific modelling and simulation, which require biventricular volumetric meshes with the right ventricle (RV) and left ventricle (LV) correctly defined from endocardium to epicardium. To this end, we conducted an evaluation of our pipeline on a 20 healthy subject dataset first presented by Andrews *et al.* [24], which includes cardiac MR and electrocardiographic imaging (ECGI) data for each subject.

## 2. Methods

### (a) Overview

**Figure 1.**
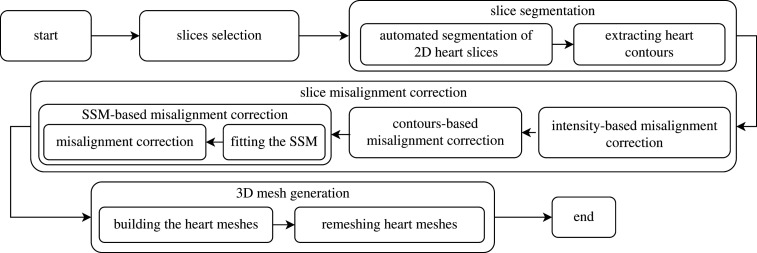
The block diagram of the proposed 3D reconstruction pipeline from multiple 2D cine MR slices.

The block diagram of the proposed pipeline is presented in figure 1. Given the acquired MR image files as the only input, the pipeline starts by automatically selecting the LAX and short axis (SAX) cardiac slices generated by standard cardiac cine MR studies. A deep learning-based automated segmentation algorithm [25] is then applied for automated extraction of the heart contours, namely, LV and RV endocardium and LV epicardium, for 3D biventricular surface modelling. The misalignment correction between slices in the 3D coordinate system is performed in a three-step process. The first two steps focus only on in-plane misalignment correction by iteratively optimizing over the intensity profiles and the intersection points between cross-sectional contours, respectively [14]. The third step performs out-of-plane misalignment corrections by optimizing over a statistical shape model (SSM). The remaining discrepancies between sparse 3D contours of intersecting slices are minimized by the surface generating algorithm, which interpolates between differing contours by orienting through the average 3D location [21]. A composition of smooth deformations aiming at maximizing fitting to image contours ensures a good matching of the reconstructed 3D biventricular surface to the input data, as well as optimal interpolation characteristics.

From the 3D biventricular surface mesh, the 3D volumetric mesh is generated using a mesh generator for electrophysiological (EP) models for the simulation of electrical activity in human heart. We have applied the automated pipeline on a cohort of 20 subjects, presented in Andrews *et al.* [24], for the generation of 3D biventricular surface and volume meshes. We have evaluated the misalignment between heart slices over the final reconstructed surface meshes, reducing average misalignment from 1.82 ± 1.60 mm to 0.72 ± 0.73 mm. Finally, we have conducted an evaluation of the ability of our generated meshes to produce physiologically coherent healthy electrical activation patterns though the computation of an Eikonal model. This second evaluation is intended to demonstrate the relevance of our method in patient-specific modelling and simulation studies of the human heart.

### (b) Proposed 3D mesh reconstruction pipeline

#### (i) Slices selection

**Figure 2.**
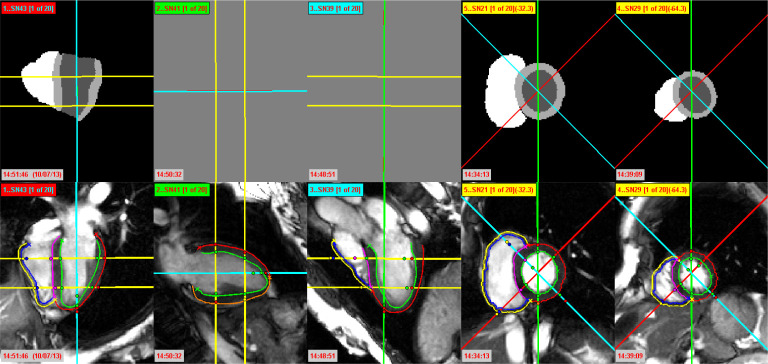
Column 1: HLA or four chamber view, column 2: VLA or two chamber view, column 3: LVOT or three chamber view, and columns 4–5: SAX slices. Top row: Automated segmentation of LV cavity (dark grey), LV myocardium (light grey), RV cavity (white) and background (black) from cine MR slices. Since we did not have an automated method for segmenting VLA and LVOT slices, those slices are missing in this figure. Bottom row: Extracted heart contours from 2D cine MR slices. The LV epicardium, LV endocardium, RV endocardium, septum and RV epicardium are represented as red, green, blue, pink and yellow contours, respectively. Since the LV epicardium or septum cannot accurately be distinguished in two chamber LAX slice (VLA view), it is separately represented as orange contour. (Online version in colour.)

The MR slices for the standard cine MRI protocol are horizontal long axis (HLA) (figure 2*a*) (also known as four chamber view), vertical long axis (VLA) (figure 2*b*) (also known as two chamber view), left ventricular outflow tract (LVOT) (figure 2*c*) (also known as three chamber view), and the stack of SAX views (figure 2*d*,*e*). In general, the cine acquisition protocol organizes the MR images according to the views, or their series names are accordingly specified in the image header files. However, for improved and robust performance irrespective of the acquisition protocol, the developed pipeline automatically identifies the SAX views according to their orientations in 3D space as stacked parallel planes; while the LAX views are identified as cross-sectional planes. Our proposed reconstruction pipeline adapts to work with any number of input image slices, even with just a small number of SAX views. However, for accurate and robust performance, we recommend to provide at least one LAX view and three SAX views, and for optimum performance, as many as possible. Since our pipeline performs intensity-based misalignment correction and contours-based misalignment correction by optimizing the similarity between LAX and SAX views, the incorporation of at least one LAX slice, along with the SAX stack, is important for the optimal misalignment correction. An example of the input cine LAX and SAX slices (for subject C12) at end-diastole is presented in the bottom row of figure 2. In figure 2, the three LAX slices, that is, HLA, VLA and LVOT, have been annotated with red, green and cyan colours, respectively; while all SAX slices are annotated in yellow. The relative position of each slice is visible on all of the other slices. The three LAX slices are presented with their respective colours on each of the SAX slices, while the yellow SAX slices are visible on all three LAX slices.

#### (ii) Slice segmentation

After selecting the cine MR slices, the pipeline next proceeds to automatically segmenting the slices and extracting the heart contours.

(A) Automated segmentation of 2D heart slices
  The biventricular surface of the heart consists of the epicardial surface and both LV and RV endocardial surfaces. In our proposed pipeline, these surfaces are reconstructed based on the epicardial contours and LV and RV endocardial contours from all of the cine MR slices. In order to identify these contours from the selected cine MR slices, we first automatically segment the heart slices into four classes: LV cavity, LV myocardium, RV cavity and background. Since the resolution of cine MR slices does not allow accurate delineation of the thin RV epicardium, we did not try to segment the RV myocardium. We have employed the deep learning-based method proposed by Bai *et al.* [25] for this step, since it has been shown to segment heart structures from cine MR slices with human-level accuracy.
  The segmentation method uses a fully convolutional network (FCN) [26], where the neural network architecture learns image features from fine to coarse scales by applying a number of convolutional filters and combines the multi-scale features for predicting the label class at each image pixel. The network is adapted from the VGG-16 network [27], where each convolution uses a 3 × 3 kernel, followed by batch normalization [28] and rectified linear unit (ReLU). After every two or three convolutions, the feature map is downsampled by a factor of 2 in order to learn features at a more global scale. Feature maps learnt at different scales are upsampled to the original resolution using transposed convolutions and the multi-scale feature maps are then concatenated. Finally, three convolutional layers of kernel size 1 × 1, followed by a softmax function, are used to predict a probabilistic label map. The segmentation is determined at each pixel by the label class with highest softmax probability. The mean cross entropy between the probabilistic label map and the manually annotated label map is used as the loss function. We have used two pre-trained networks for segmenting the SAX slices and the four chamber LAX (HLA view) slices in our pipeline. Both networks were originally trained over more than 3750 subjects from the UK Biobank Study [29], with manual annotations of LV endocardial and epicardial borders and the RV endocardial borders at end-diastolic and end-systolic time frames. Data augmentation was performed on-the-fly, which applied random translation, rotation, scaling and intensity variation to each mini-batch of 20 image slices, before feeding them to the network. The Adam method [30] was used for optimizing the loss function, with a learning rate of 0.001 and iteration number of 50 000. Complete details of the network architecture are available at [25].
  Since the FCN can automatically segment the LV from all heart phases of a standard cine MR acquisition, we have applied it over the whole cardiac sequence and measured the LV volume from the segmented SAX stack. Since the heart ventricles are most expanded at the end-diastolic phase, the end-diastole is automatically identified as the cardiac phase with maximum LV volume. The end-systole is similarly identified as the cardiac phase with minimum LV volume.
  Since we have used both pre-trained networks in our pipeline without any additional re-training on our dataset, their performance can be affected. Although the method works well for segmentation of SAX slices, it can produce suboptimal results in four chamber LAX slices. Hence, our pipeline includes an additional optional step for *Manual Contouring of Heart Slices*. After automatically extracting the LV and RV endocardial and LV epicardial contours from the segmented slices, the method allows manual adjustments of contours, if necessary, on a graphical interface. Since at this time we do not have a pre-trained network for the VLA view and LVOT view slices, we have manually contoured the LV and RV endocardial and LV epicardial surfaces on the LVOT view slices and LV endocardial and epicardial surfaces on the VLA view slices. An example of automated segmentation of the four chamber LAX and the SAX cine MR slices (visible in the bottom row of figure 2) for subject C12 is presented in the top row of figure 2.
(B) Extracting heart contours
  After segmentation, we automatically identify all necessary heart contours and extract them for 3D reconstruction. In SAX slices, the LV endocardial contours (represented as green contours in the bottom row of figure 2) should always be closed. The septal wall for both LAX and SAX slices is identified as the intersection between LV epicardium and RV endocardium (represented in pink in the bottom row of figure 2), while the remaining contours’ segments are identified as the LV epicardium and RV endocardium (represented as red and blue contours, respectively). Since the RV epicardium cannot be resolved at the cine MR resolution, we synthesized RV epicardial contours by applying a uniform wall thickness of 3 mm (represented as yellow contours in the bottom row of figure 2) on the RV endocardium, based on an *ex vivo* study of human hearts [31].

#### (iii) Slice misalignment correction

**Figure 3.**
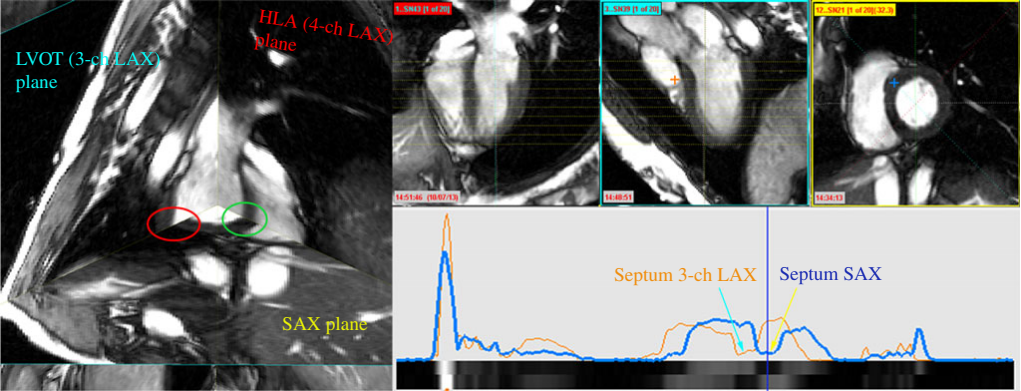
Left: Cine MR slices in their 3D spatial positions. Here, a good alignment exists between HLA and SAX (in green ellipse); but a clear misalignment is visible between LVOT and SAX (in red ellipse). Right: At the top, the same three HLA, LVOT and SAX slices, and at the bottom, the intensity profiles of LVOT (in orange) and SAX (in blue) along their intersection line, where a misalignment at the septum is visible. The blue vertical line along the profile corresponds to the location along the intersection line (denoted by ‘+’) on the 2D slices above. (Online version in colour.)

**Figure 4.**
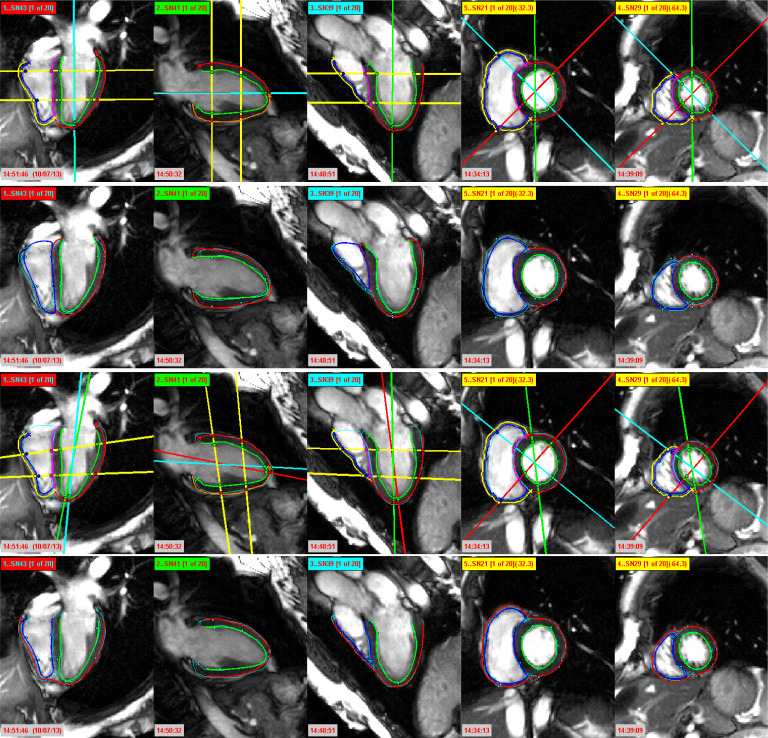
Row 1: The heart contours on 2D cine MR slices, after contours-based misalignment correction. The coloured dots represent the position of the corresponding contours on the intersecting slice. Row 2: The reprojection of the fitted statistical shape model on 2D cine MR slices. The red, green and blue contours, respectively, denote the LV epicardium, LV endocardium and RV endocardium of the shape model. Row 3: Optimal misalignment correction using the fitted statistical shape model. Row 4: The reprojection of the reconstructed 3D biventricular mesh on 2D cine MR slices. (Online version in colour.)

**Figure 5.**
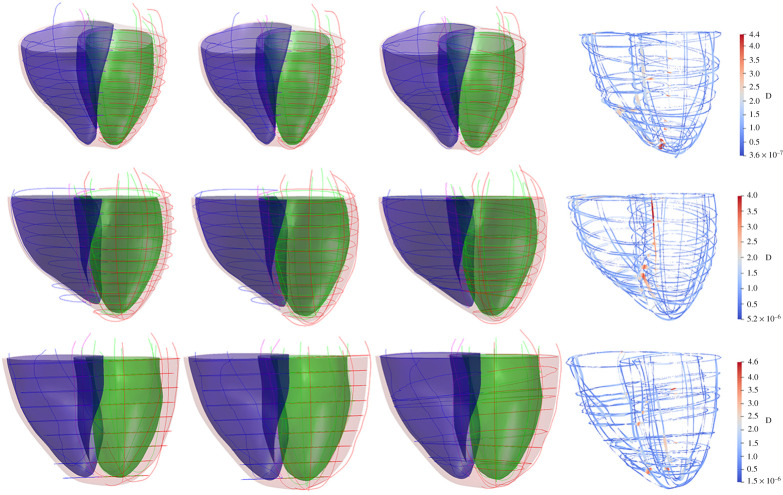
Left to right: The initial sparse misaligned contours, the contours after contours-based misalignment correction, the contours after SSM-based misalignment correction, and the distances of corrected contours from the final mesh represented using a colour map. In the first three columns, the reconstructed mesh is shown for reference, with LV and RV endocardium in green and blue, respectively, and the epicardium in red. Each mesh is cropped at the level of the most basal SAX contoured. Three representative cases (subjects C2, C10 and C12) are shown in the three rows. (Online version in colour.)

After extracting the heart contours from 2D cine MR images, the pipeline proceeds to orienting the heart slices in 3D space and correcting for possible misalignments. It consists of three consecutive steps: (A) intensity-based misalignment correction, (B) contours-based misalignment correction and (C) statistical shape model (SSM)-based misalignment correction. The first two-steps perform only in-plane misalignment corrections, while the third step performs mainly out-of-plane misalignment corrections.

(A) Intensity-based misalignment correction of heart slices
  Our first step for misalignment correction performs the registration of heart slices in 3D space based on the image intensity profiles along the common line between intersecting slices [14]. The aim of this step is to provide spatial consistency to the slices in 3D space, by comparing the intensity profiles at the line formed by the intersection between two slices. As per [14], we follow the assumption that two slices will align perfectly when the underlying features of the line profile at their intersection match. In this step, we only assume that slices from a subject are triggered at the same cardiac phase and the 3D shape of the heart remains fixed among slices; and hence, only rigid-body transformations between slices are considered. An example of the misalignment between cine MR slices in 3D space is presented in figure 3.
  We measure the global motion (GM) discrepancy of the slices as the sum of (dis)similarity measures *E* between a pair of intersecting slices [14]. Let the *i*th slice be *S*_*i*_ and the set of its rigid transformation parameters be $\Theta_{i}$, where $\Theta =\{t_{x},t_{y},t_{z},R_{\alpha },R_{\beta },R_{\gamma }\}$, that is, the 3 translations and 3 Euler angles. The rigid transformation of the slice *S*_*i*_ by $\Theta_{i}$ is defined as $S_{i}^{\Theta_{i}}$. The GM discrepancy is then given by
  2.1 $$\text{GM}(\Theta_{1},\ldots ,\Theta_{n};S_{1},\ldots ,S_{n})=\sum_{i,j}E(S_{i}^{\Theta_{i}},S_{j}^{\Theta_{j}}).$$
  Here, the summation operation contains all pairs of slices *S*_*i*_ and *S*_*j*_ that intersect. The objective is to minimize *GM* by estimating the parameters of the optimal rigid transformation Θ for each slice. Since *GM* is defined as a sum of dissimilarity measures *E*, we have adopted an iterative minimization of partial terms, where, in each iteration, we sequentially optimize the rigid transformation Θ for a single slice while keeping the others fixed.
  Due to the high-intensity disparity between image slices, we have used feature-based similarity measures, instead of intensity difference-based measures, for robust assessment of similarity at the intersection profiles. As local phase is independent of contrast and not affected by intensity inconsistencies, we have used it as a similarity measure. Although our registration is based on the similarity between the 1D intersecting line profiles, we have computed the local phase for an entire image, and then obtained the line intersection between two local phase images in order to reduce noise along the line profiles [32]. In 2D, the local phase information is obtained by convolving the images with banks of quadrature pairs of log-Gabor filters [33]. After calculating the local phase images, the line intersection at both images is taken into account to compute the normalized cross correlation (NCC), as the final similarity measure between two profiles. For robust and faster performance, we first performed the iterative registration of only LAX slices in our implementation, and then included all of the LAX and SAX slices for the final registration.
  Although the following step of contours-based misalignment correction tries to solve the same problem as the intensity-based misalignment correction, the intensity-based approach is useful when the segmentations of individual slices are not provided. In addition, the application of intensity-based misalignment correction generally improves the robustness of the subsequent contours-based misalignment correction and improves the accuracy of overall in-plane misalignment correction by approximately 5–15%.
(B) Contours-based misalignment correction
  Given the heart contours, it is possible to optimally align the LAX and SAX heart slices in 3D space to minimize the misalignments. However, due to motion-related deformations, inconsistencies in contouring of the slices, and the transformation of 2D slices to 3D space based on their in-plane resolution, a perfect alignment, where the distances between LAX and SAX contours are zero, is usually not achievable. Hence, in order to achieve the optimum alignment of the LAX and SAX contours, we have applied an iterative misalignment correction algorithm using the extracted heart contours. The applied algorithm in this step is identical to the misalignment correction algorithm used in §2b(iii)(A), except in one key step. Instead of using NCC over local phase images as the dissimilarity measure *E*( · ) in equation (2.1), here we have used the Euclidean distance between LAX and SAX contours. We have first performed the iterative alignment of only LAX slices, and then included all of the LAX and SAX slices for the final misalignment correction. The misalignment correction of the cine MR slices from the bottom row of figure 2 is presented in the top row of figure 4. Note that, after the misalignment correction, the intersection points between planes (represented as coloured dots) are now very close to each other and they often coincide. Hence, with this step, we generate a sparse 3D representation of the true biventricular surface, as visible in column 2 of figure 5.
(C) Statistical shape model-based misalignment correction
  Although the contours-based misalignment correction can provide a good alignment of the cine MR slices, some residual misalignment may still persist. Since the optimum misalignment correction based on the intersections between sparse LAX and SAX slices is an ill-posed problem and hence can produce undesirable solutions, we have optimized the first two steps for the misalignment correction based only on the in-plane rigid transformations. However, one major cause of misalignment, the motion artefacts between image acquisitions, cause out-of-plane movements. Hence, our objective in this step is to estimate the optimal alignments of the cine slices, including both in-plane and out-of-plane alignments, in 3D for accurate surface reconstruction. The overall procedure can be split into two steps.
  — Fitting the statistical shape model
    In order to minimize the misalignment artefacts, specifically the out-of-plane misalignments, we first fit a statistical shape model (SSM) to the misaligned heart contours. To this end, we have employed an SSM of human heart ventricles by Bai *et al.* [34,35]. The SSM was created by registering 1093 hearts, generated by high-resolution MR images, to the template space using rigid registration, thus removing the position and orientation differences, and then by applying the principal component analysis on the surface meshes of LV, RV and both ventricles. The first 100 principal components, which account for 99.9% of the shape variation, and the mean SSM are publicly available at http://wp.doc.ic.ac.uk/wbai/data/.
    For our misalignment correction, the surface meshes are fitted to the sparse heart contours in 3D space by the optimal estimation of the 100 principal components of the SSM, followed by rigid transformation. The reprojection of the fitted SSM over the cine slices from the top row of figure 4 is presented in the second row of figure 4.
  — SSM-based misalignment correction
    The objective of fitting the SSM is to provide a reference for the alignment of heart contours of the cine slices. In our previous step (§2b(iii)(B)), we performed the misalignment correction using only intersection points between LAX and SAX slices. In order to remove the remaining misalignment, we have applied rigid transformations to optimally align the heart contours to the fitted SSM. The process is sequentially performed for all LAX and SAX slices. The results of this step using the fitted SSM is presented in the third row of figure 4.
    It should be noted that the sole objective of the SSM is for guiding a robust misalignment correction of the heart contours. Hence, after this step we discard the fitted shape model and proceed only with the aligned heart contours for 3D surface mesh reconstruction. Since the SSM-based misalignment correction mainly focuses on out-of-plane misalignment correction, the application of intensity-based and in particular the contours-based misalignment correction is of utmost importance for in-plane misalignment correction. Quantitatively, the prior two-steps for in-plane misalignment correction improve the performance of SSM-based misalignment correction by approximately 30–40%.

#### (iv) 3D mesh generation

(A) Building the heart meshes
  After the final alignment of the contours, 3D surface meshes are generated for each of LV endocardium, RV endocardium and epicardium, using the method proposed by Villard *et al*. [20,21]. The method can generate a 3D surface mesh from sparse, heterogeneous, non-parallel, cross-sectional, non-coincidental contours. The initial meshes are created connecting the contours for each of the three surfaces. Depending on the availability of the LAX information, the algorithm initializes the apex as the lower bound mesh point from the lowest contour points in that plane, using the LAX information. Analogously, the upper mesh point, representing the most basal slice, is initialized as the mean position of the topmost contour points lying on the plane that contains such point. The meshes are then fitted to the contours using a thin plate splines algorithm [36,37]. The algorithm deforms the initial mesh iteratively towards the heart contours, resulting in a smooth approximation. The independent reconstruction of three surface meshes often leads to an undesirable intersection of epicardial and endocardial surfaces, especially in the thin RV. We separate the intersecting surfaces using an adaptation of the attractor-based mesh deformation algorithm [21]. Attractor points are added near the intersection between two surfaces. Epicardial surface points that lie on the inside of the endocardial surface become attractor points for the endocardial surface and vice versa. In case this process cannot separate the surfaces after two iterations over all intersecting points, the endocardial surface still lying on the outer side of the epicardial wall becomes the epicardial surface. Equivalently, the reverse happens when the epicardial surface lies on the inner side of the endocardial wall. Laplacian smoothing, decimation, and affine transformation are applied to ensure that the new surface patches are well embedded in the surfaces. Finally, the three meshes are joined together at the basal plane to generate the biventricular mesh. The reprojection of the reconstructed 3D biventricular mesh using the aligned contours on cine slices from third row of figure 4 is presented in the bottom rowof figure 4.
(B) Remeshing heart meshes
  To produce the final reconstructed 3D biventricular heart surface, the biventricular mesh generated in the previous step is remeshed with a restricted Frontal–Delaunay algorithm using the mesh generator JIGSAW (http://github.com/dengwirda/jigsaw) [38]. Since the majority of biophysical electrophysiological models are solved using the finite element method with unstructured meshes of tetrahedral elements, TETGEN (http://wias-berlin.de/software/tetgen/) [39] is used on the remeshed 3D surface to build the final tetrahedral mesh. In all our experiments, we have specified the element size for both generators as 1.5 mm.

All the algorithms and measures in the current study are implemented in Matlab R2014a (The Mathworks, Inc.) and executed in Windows 10 64-bit OS machine with an Intel(R) Xeon(R) CPU E5-1650 v3 at 3.50 GHz and 32 GB RAM. The initial step of loading and selecting cine MR slices in the pipeline takes 2–2.5 min, while the second step of slice segmentation takes around 2 min. The third step of misalignment correction is split into three steps. The first two steps focus on in-plane misalignment correction based on the intensity information and heart contours, and take on average 4 min and 10 min, respectively. The third step of SSM-based out-of-plane misalignment correction is relatively time-consuming. The first sub-step fits an SSM to the misaligned contours in 1–1.5 h of time, while the second sub-step aligns the contours with respect to the fitted SSM in 2.5–3 h. The final step of the proposed pipeline generates a surface mesh and a tetrahedral mesh over the aligned contours in 30–45 min. Therefore, in total, the proposed pipeline can take 5–5.5 h to prepare a biventricular mesh of optimal quality given the cine MR slices. However, with the assumption of no out-of-plane misalignment between cine MR slices, the SSM-based misalignment correction step can be skipped, reducing the average run-time of the proposed pipeline to 1 h.

### (c) Evaluation of the pipeline

#### (i) Dataset

We have evaluated the reconstruction performance of the proposed automated pipeline on a cohort of 20 healthy adults enrolled at Washington University in St. Louis [24]. The study was approved by the Human Research Protection Office at Washington University in St Louis. All participants provided written informed consent. MRI scans were performed using a 1.5T scanner (MAGNETOM Avanto, Siemens Medical Solutions, Malvern, PA). Before the scan, the ECGI recording electrodes were replaced with MRI-visible markers so that the electrode positions could be obtained in the same coordinate system as the heart geometry. A navigated anatomic sequence was used so the electrode positions and cardiac anatomy data could be localized in the same coordinate frame.

**Figure 6.**
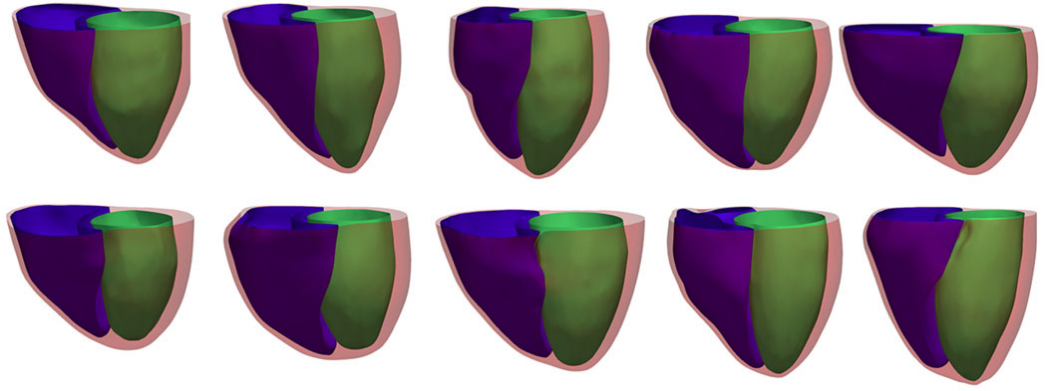
The reconstructed 3D biventricular meshes from 10 subjects. The green, blue and red surfaces, respectively, denote the LV endocardium, RV endocardium and the epicardium. (Online version in colour.)

A series of SAX image sets were obtained in parallel planes at 8 mm intervals beginning at the level of the mitral valve and ending at an imaging plane that contained only apical myocardium. Additionally, four sets of radially oriented LAX images, separated by 45° and intersecting at the centroid of the LV, were obtained. For each selected imaging plane, a 2D balanced steady-state free precession cine image acquisition was collected. Both SAX and LAX MR images were ECG-gated beginning at end-diastole. The images were acquired for a complete cardiac cycle during the same breath hold to ensure similar anatomic positioning. The number of SAX cardiac cine MR slices varied between 9 and 14, while the number of acquired frames over a cardiac cycle varied between 17 and 27. All images have in-plane resolution of 1.3672 mm. For the 3D reconstructions in the current study, we have selected the end-diastolic phase, identified by the first image frame according to the acquisition protocol, from all cine MR acquisitions. A selection of 10 reconstructed heart surfaces from our electrophysiology study is presented in figure 6.

#### (ii) Evaluating the segmentation performance

In order to evaluate the performance of the automated segmentation method discussed in §2b(ii), we have applied the segmentation method on a randomly selected sample of 20 subjects from the UK Biobank cohort [29] and compared it with the corresponding ground-truth segmentations annotated by a trained individual. The quantitative performance analysis is presented in table 1 with respect to the Dice coefficient and Hausdorff distance. The quantitative performance analyses of the segmentation results have also been performed for the dataset of our current study [24] and the results are reported in table 2 with respect to both Dice coefficient and Hausdorff distance.

**Table 1.** The performance of automated segmentation over 20 subjects randomly selected from the UK Biobank dataset.

	SAX stack		four chamber LAX	
segment	Dice	Hausdorff (pixel)	Dice	Hausdorff (pixel)
LV cavity	0.9678 ± 0.0196	1.17 ± 0.30	0.9796 ± 0.0059	1.64 ± 0.60
LV myocardium	0.8725 ± 0.0595	2.10 ± 2.32	0.9271 ± 0.0211	1.93 ± 0.88
RV cavity	0.9363 ± 0.0547	2.29 ± 2.35	0.9621 ± 0.0164	2.42 ± 1.24

**Table 2.** The performance of automated segmentation over 20 subjects in the dataset of our electrophysiology study.

	SAX stack		four chamber LAX	
segment	Dice	Hausdorff (pixel)	Dice	Hausdorff (pixel)
LV cavity	0.9096 ± 0.1811	3.74 ± 5.10	0.7841 ± 0.1858	20.77 ± 13.18
LV myocardium	0.7815 ± 0.1945	5.80 ± 7.88	0.5932 ± 0.2352	22.90 ± 13.98
RV cavity	0.6799 ± 0.2825	13.98 ± 10.70	0.4891 ± 0.3213	28.13 ± 12.76

#### (iii) Evaluating the misalignment correction

**Figure 7.**
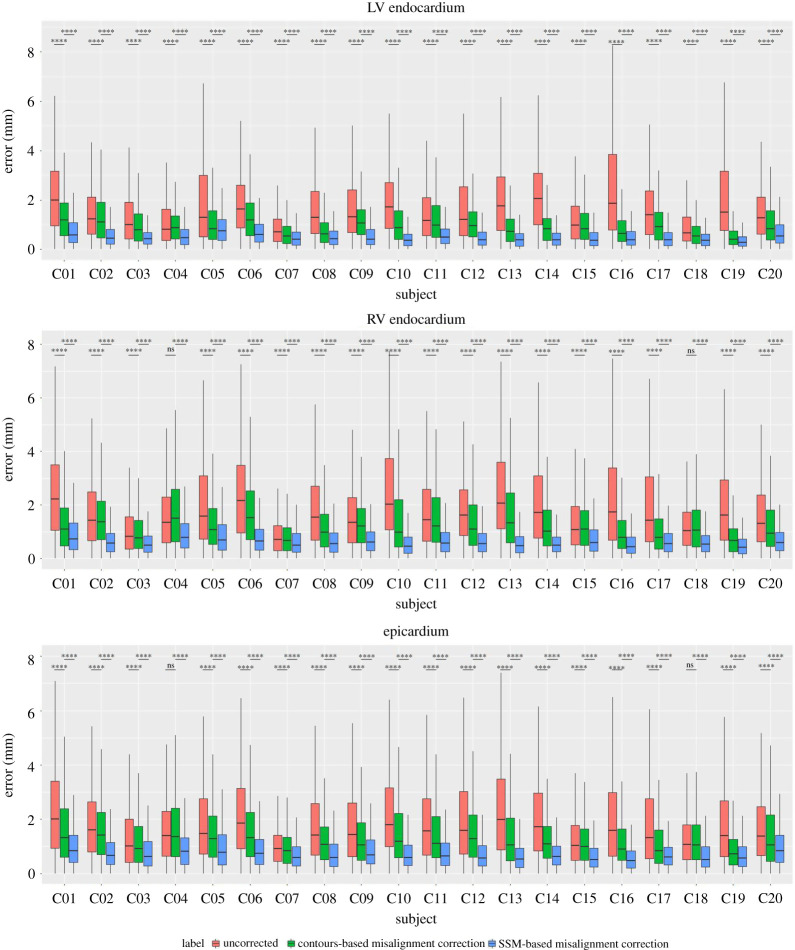
Box-plots for the evaluation of contours-based and statistical shape model-based misalignment corrections. The ‘****’ and ‘ns’ symbols are used to indicate *p*-value of less than 0.0001 and non-significant *p*-value, respectively. (Online version in colour.)

The cine MR slices, and hence heart contours, presented in column 1 of figure 5, are severely affected by the motion artefacts between image acquisitions. The qualitative performance of contours-based misalignment correction is presented in column 2 of figure 5. The corresponding quantitative analysis is presented in figure 7 separately for LV endocardium, RV endocardium and epicardium, by computing the distances of sparse heart contours from the final reconstructed heart surfaces. After the contours-based optimal misalignment correction, we have applied our automated method for out-of-plane misalignment correction using a statistical shape model. The qualitative performance of misalignment correction using SSM over 3D sparse heart contours is presented in column 3 of figure 5. The quantitative validations are presented for LV endocardial, RV endocardial and epicardial contours on the same figure 7.

We have assessed the performance of both contours-based and SSM-based misalignment corrections using the paired-*t* test (upper-tailed), by testing for a significant reduction in average (population) mean for the LV endocardium, RV endocardium, and epicardium of each subject. In order to compare the performance of both contours-based and SSM-based misalignment corrections over all 20 subjects, we have adopted the linear mixed model analysis, with Kenward and Roger’s method applied for the degrees of freedom of the *t*-statistic [40]. The different subjects are included as random effects in the linear mixed model.

#### (iv) Evaluating the mesh characteristics

In order to evaluate the quality of our reconstructed meshes, we have computed the Knupp index, measured by the scaled Jacobian for each element of each mesh, to find a measure of the distortion of the tetrahedra [41–43]. A Knupp index close to 1 indicates a perfectly regular tetrahedron, while one close to 0 indicates a highly distorted tetrahedron.

#### (v) Electrophysiological simulations using the biventricular meshes

To demonstrate that our automated 3D reconstruction method enables subject-specific modelling and simulation, we combine the 3D cardiac meshes from four subjects in the dataset with the Eikonal model, for simulation of electrical excitation through the ventricles. The Eikonal model enables the computation of the activation times at each point in the ventricular mesh, producing an activation time map for each subject.

The electrical model is personalized for each subject based on the experimental epicardial activation time maps obtained using ECGI, as described in Andrews *et al.* [24]. The ECGI-derived epicardial activation time maps were obtained in a 3D surface mesh of lower resolution than the ones generated with our pipeline. Valve plane regions, which were excluded from the original ECGI analysis, were removed. Next, we aligned our 3D CMR-based volumetric mesh with the 3D low-resolution surface mesh provided with the ECGI reconstructions in the dataset and mapped the epicardial activation map to our 3D generated mesh using nearest-neighbour.

We then adopted the inference methodology presented in Camps *et al.* [44] to calibrate the electrophysiological model properties to yield simulated activation time maps in agreement with the ECGI-based experimental ones. More precisely, the inference method estimates the human ventricular activation properties, namely, endocardial sites of first activation, orthogonal myocardial tissue electrical conduction speeds, and endocardial isotropic conduction speed, from epicardial activation time maps and the subject-specific CMR-based 3D geometry. Moreover, we constrained the conduction property values to be within the ranges reported in the human electrophysiological literature [45].

## 3. Results

### (a) Evaluation of segmentation accuracy

From the segmentation results presented in table 1 for 20 subjects randomly selected from the UK Biobank cohort, we can observe that the automated segmentation method can segment the SAX and four chamber LAX slices accurately for all three regions of interest. Combined over the 20 subjects, the average Dice scores for the LV cavity, LV myocardium and RV cavity are 0.9678, 0.8725 and 0.9363, respectively, in the SAX stack and 0.9796, 0.9271 and 0.9621, in the four chamber LAX. Hence, based on this analysis, we can state that the segmentation method in our proposed pipeline can automatically deliver the segmentation of LV cavity, LV myocardium and RV cavity to near human-level accuracy from the four chamber LAX and SAX stack, when the standard cine MR image acquisition protocol is maintained.

Due to the simultaneous generation of tagged and non-tagged MR slices for the study with electrocardiographic imaging, the acquisition protocol of the electrophysiology study [24] did not capture the cine images, specifically the LAX slices, in the standard acquisition planes for a cine MR study. It acquired four sets of radially oriented LAX images, separated by 45° and intersecting at the centroid of the LV, for each patient and did not capture the image at the standard four chamber LAX plane. Hence, our segmentation network, trained over more than 3750 subjects, did not perform optimally on this dataset. From the quantitative indices reported in table 2, we can observe that our pretrained segmentation network did not provide a satisfactory segmentation performance for the four chamber LAX. Although the performance is relatively better for the segmentation of LV cavity with respect to the Dice coefficient (0.7841 ± 0.1858), it is not satisfactory for the segmentation of LV myocardium (with Dice 0.5932 ± 0.2352) and poor for RV cavity (with Dice 0.4891 ± 0.3213). For the SAX stack, the segmentation network performed well for the LV cavity and LV myocardium. The average Dice coefficient and Hausdorff distance for the SAX stack over 20 subjects are reported as 0.9096 ± 0.1811 and 3.74 ± 5.10 pixels, respectively, for the LV cavity and 0.7815 ± 0.1945 and 5.80 ± 7.88 pixels, respectively, for the LV myocardium. For the RV cavity in SAX stack, the network provided suboptimal performance with Dice 0.6799 ± 0.2825. Since the segmented heart contours are the primary inputs for the subsequent misalignment correction and mesh reconstruction steps, accurate delineation of these contours is of utmost importance. Hence, in order to compensate for the suboptimal segmentation performance in the dataset of the electrophysiology study, the manually adjusted contours have been used for subsequent evaluation steps of the electrophysiology study.

### (b) Evaluation of misalignment correction

From the quantitative results presented in figure 7, we can observe that the contours-based misalignment correction method has been able to reduce the misalignment of heart contours from the final surface mesh from average 1.82 ± 1.60 mm to average 1.28 ± 1.15 mm. The misalignment error has been reduced from 1.70 ± 1.57 mm to 1.03 ± 0.89 mm for the LV endocardial contours, from 1.87 ± 1.60 mm to 1.34 ± 1.18 mm for the RV endocardial contours, and from 1.86 ± 1.61 mm to 1.40 ± 1.24 mm for the epicardial contours. For most of the subjects, the results are statistically significant in LV endocardium, RV endocardium, and epicardium, with respect to the parametric paired-*t* test (upper-tailed), where 0.05 is the desired level of significance. Only for the subjects C4 and C18, the contours-based misalignment correction cannot produce statistically significant performance in the RV endocardium and the epicardium. Using the linear mixed model analysis over all 20 subjects, we have found that the contours-based misalignment correction achieves statistically significant performance over the uncorrected slices for the LV endocardium, RV endocardium and epicardium.

After the contours-based misalignment correction, the misalignment correction method using the statistical shape model has been able to reduce the misalignment error from average 1.03 ± 0.89 mm to average 0.56 ± 0.47 mm for the LV endocardial contours, and from average 1.34 ± 1.18 mm to average 0.68 ± 0.55 mm for the RV endocardial contours, as presented in figure 7 for 20 subjects. For the epicardial contours, the SSM has been able to reduce the misalignment error from average 1.40 ± 1.24 mm to average 0.84 ± 0.92 mm. For all of the subjects, the misalignment reductions using the SSM-based approach are statistically significant with respect to the parametric paired-*t* test (upper-tailed). Using the linear mixed model analysis over all 20 subjects, we have evaluated that the SSM-based misalignment correction achieves statistically significant performance over the contours-based misalignment correction for the LV endocardium, RV endocardium and epicardium.

### (c) Evaluation of mesh characteristics

**Figure 8.**
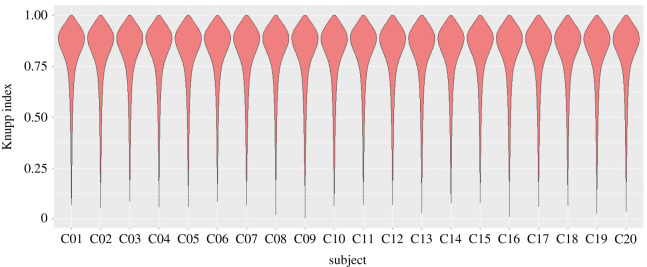
Violin plots of the Knupp index for the evaluation of mesh characteristics of 20 subjects. (Online version in colour.)

Table 3 presents the average values of the Knupp index, along with the standard deviations, in order to demonstrate the quality of our 20 reconstructed meshes. It also reports the number of nodes and the number of elements in each mesh. From the results presented in the table, we can infer that our proposed pipeline can produce robust and high-quality tetrahedral meshes, with the average Knupp index lying between 0.79 and 0.81. We have also illustrated the distribution of the Knupp index for all 20 subjects using violin plots in figure 8. The positively skewed distributions of the violin plots with mode around 0.9 and the thin tails below the threshold of 0.5 for all subjects indicate that our pipeline can generate robust and high-quality tetrahedral meshes.

**Table 3.** Characteristics of 20 reconstructed tetrahedral meshes.

subject	# nodes	# elements	Knupp index	subject	# nodes	# elements	Knupp index
C1	132397	747110	0.7989 ± 0.1559	C11	151391	839070	0.8015 ± 0.1542
C2	112075	615361	0.8017 ± 0.1546	C12	108534	610495	0.7963 ± 0.1578
C3	118682	664708	0.8010 ± 0.1543	C13	165238	966925	0.7953 ± 0.1596
C4	112692	625434	0.8018 ± 0.1538	C14	131131	741295	0.7956 ± 0.1582
C5	210675	1202873	0.7999 ± 0.1558	C15	137451	773999	0.8013 ± 0.1545
C6	128958	715692	0.8051 ± 0.1512	C16	151392	864484	0.7936 ± 0.1621
C7	116844	653490	0.7964 ± 0.1587	C17	107294	596422	0.7970 ± 0.1585
C8	168373	949463	0.8017 ± 0.1547	C18	121949	670831	0.7994 ± 0.1553
C9	126457	719521	0.7941 ± 0.1611	C19	218982	1298886	0.7948 ± 0.1605
C10	130336	726214	0.7943 ± 0.1607	C20	199500	1155478	0.7970 ± 0.1589

### (d) Evaluation of the ability to reproduce realistic activation sequences

**Figure 9.**
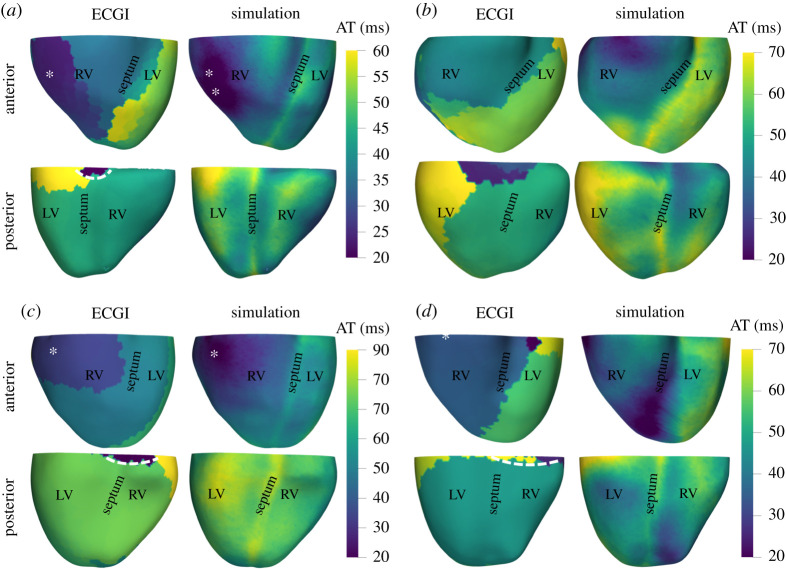
Reproduction of activation maps for subjects (*a*) C2, (*b*) C3, (*c*) C8 and (*d*) C12. For each subfigure, left, sinus rhythm activation time (AT) ECGI map with epicardial breakthrough in the RV (asterisk) and valve plane artefacts (dashed lines); right, Eikonal simulated activation map after running parameter inference. Top rows are the anterior views, and bottom the posterior views. Pearson’s correlation coefficient between ECGI and simulated epicardial activation times, excluding ECGI-related artefacts: (*a*) C2 – 0.88; (*b*) C3 – 0.68; (*c*) C8 – 0.91 and (*d*) C12 – 0.79. (Online version in colour.)

Figure 9 illustrates the reproduction of the ECGI-derived activation maps for subjects C2, C3, C8 and C12, in subfigures A, B, C and D, respectively, together with their corresponding reproduction accuracy. The reproduction accuracy was computed using Pearson’s correlation coefficient between the epicardial activation time values derived from the ECGI solution and those simulated from the inference method presented in [44].

This inference method [44] combined with the patient-specific mesh were able to calibrate the Eikonal model to reproduce the electrical activity reported in the dataset through ECGI (figure 9). Overall, we observed that our simulations reproduced consistently the patterns in the activation sequences (figure 9) measured in healthy subjects [24]. For example, the epicardial wave breakthroughs (marked with asterisks in the figure) have been correctly identified in the cases where it was visible from the ECGI activation map, namely, for subjects C2 (figure 9*a*) and C8 (figure 9*c*). As we had anticipated, the small differences between our simulations and the ECGI-derived activation maps were located near the valve plane due to the removal of valve plane signals containing artefacts (white dashed lines in figure 9*c*,*d*) from the data used for the inference.

## 4. Discussion

The 3D reconstruction of heart meshes from 2D cine MR slices is important for several clinical reasons. Although the 2D MR images can exhibit the presence of scar or oedema, or myocardial perfusion on heart structures, their visualization on the 3D heart shape can help in the understanding of their locations and shapes. Quantification of heart shape has been shown to improve diagnosis and prediction of patient outcome. This shape can only be fully described in 3D. The reconstruction of 3D heart shape is also important in 3D printing applications that represent a growing field [46,47]. To this end, here we have proposed a completely automated approach for reconstructing 3D heart meshes from 2D cine MR slices.

The advantage of our proposed method is that it can easily be integrated in existing diagnostic systems in the clinical environment. The proposed method performs in an end-to-end automated way, where it can generate the final reconstructed meshes given only the image files. Our method automatically selects the necessary cine MR images for reconstruction, segments them, optimally corrects for the misalignment between heart slices, and finally generates the surface as well as volumetric 3D biventricular meshes. Since our method is completely automated, it can perform without any expert supervision. However, in case the user requires to interact and improve the accuracy of the automatically extracted heart contours derived from automated segmentation, an optional step for manual adjustment through a graphical interface is provided inside our pipeline.

For optimally removing the misalignment between heart slices, the pipeline incorporates three methods (§2b(iii)). Among these three steps, the most important is the contours-based misalignment correction. This automated step uses an iterative algorithm to optimally reduce the distances between extracted contours on heart slices in 3D space using rigid transformations. The prior intensity-based misalignment correction mainly provides a good initial alignment of heart slices for this step. With the application of this step, we have been able to reduce the misalignment between heart slices to 1.28 ± 1.15 mm in our experiment over 20 healthy subjects. Since the cine MR images included in our study have in-plane resolution of 1.3672 mm, we consider this performance adequate for our reconstruction purpose. However, for optimum performance, we recommend to incorporate the additional step of misalignment correction using the statistical shape model. With the help of a fitted SSM in this step, the pipeline has been able to resolve out-of-plane misalignment between slices and reduced the misalignment to 0.72 ± 0.73 mm in our experiment over 20 subjects. In order to optimally demonstrate the performance of our proposed reconstruction pipeline, we have provided a supplementary multimedia file, where the selection of cine MR slices, extraction of sparse heart contours, intensity-based misalignment correction, contours-based misalignment correction, SSM-based misalignment correction and the final reconstructed 3D meshes are presented step-wise for all 20 subjects.

A key feature of our biventricular meshes is their relevance for modelling and simulation-based applications, such as the digital twin [3] and *in silico* clinical trials [48], that account for the effect of anatomical variability in the human population [5]. Our patient-specific meshes paired with the selected inference method [44] demonstrated being capable to reproduce activation sequences consistent with those observed from the subjects’ ECGI recordings. More precisely, the inference method has been able to calibrate the Eikonal model to reproduce most of the patterns and behaviours observed from the activation maps obtained through ECGI. For example, our simulations successfully replicated all epicardial wave breakthroughs (see white asterisk in figure 9) present in the ECGI reconstructions while omitting the small valve plane artefacts (white dashed lines in figure 9). The values for the activation properties inferred through this process were consistent with human electrophysiological literature [45] (see electronic supplementary material). This ability to reproduce the physiological patterns in the ECGI activation maps demonstrates that our anatomical reconstructions of the heart are relevant for applications such as *in silico* clinical trials or digital twin applications towards the realization of precision medicine.

With the recent developments in 3D cine MR acquisitions [49–51], the proposed pipeline can also be adapted to reconstruct 3D biventricular meshes from 3D cine MR scans. Due to the high image resolutions of the 3D cine MR imaging, the misalignment correction step will not be necessary. The pipeline can be applied to first perform the automated segmentation for extracting the relevant heart contours, and then the mesh reconstruction step will be applied for automated reconstruction of the 3D biventricular meshes. In our future work, we wish to extend our 3D biventricular mesh reconstruction pipeline to include the automated 3D reconstruction of both atria from the same 2D cine MR acquisitions. In addition, we also wish to incorporate the automated 3D representation of tissue heterogeneities on our final reconstructed meshes to serve in clinical decision-making for risk stratification and for personalized simulation studies in patients displaying scars or fibrotic patches in the heart.

## Data accessibility

The input images in our study (cardiac MRI and ECGI) contain patient data and, hence, they cannot be shared in a public domain. The results of this study such as the reconstructed 3D surface and volumetric meshes and the motion-corrected 3D sparse contours can be obtained from the corresponding author upon reasonable request. In order to demonstrate the efficacy of the proposed reconstruction pipeline, a montage of the analysis over all 20 subjects is provided as the electronic supplementary material video file. The video sequentially illustrates the input cardiac cine MR images, the extracted sparse heart contours, the intensity-based, contours-based and statistical shape model-based misalignment corrections, and finally the reconstructed 3D biventricular meshes for all 20 subjects in 360° 3D rotating frames. Additional information such as implementation details of the reconstruction pipeline, electrophysiological inference results, and detailed qualitative results of each step of the proposed reconstruction pipeline are provided in the electronic supplementary material ‘Appendix’ file.
